# Na^+^-stimulated ATPase of alkaliphilic halotolerant cyanobacterium *Aphanothece halophytica *translocates Na^+ ^into proteoliposomes via Na^+ ^uniport mechanism

**DOI:** 10.1186/1471-2091-11-30

**Published:** 2010-08-07

**Authors:** Kanteera Soontharapirakkul, Aran Incharoensakdi

**Affiliations:** 1 Laboratory of Cyanobacterial Biotechnology, Department of Biochemistry, Faculty of Science, Chulalongkorn University, Bangkok 10330, Thailand

## Abstract

**Background:**

When cells are exposed to high salinity conditions, they develop a mechanism to extrude excess Na^+ ^from cells to maintain the cytoplasmic Na^+ ^concentration. Until now, the ATPase involved in Na^+ ^transport in cyanobacteria has not been characterized. Here, the characterization of ATPase and its role in Na^+ ^transport of alkaliphilic halotolerant *Aphanothece halophytica *were investigated to understand the survival mechanism of *A. halophytica *under high salinity conditions.

**Results:**

The purified enzyme catalyzed the hydrolysis of ATP in the presence of Na^+ ^but not K^+^, Li^+ ^and Ca^2+^. The apparent K_*m *_values for Na^+ ^and ATP were 2.0 and 1.2 mM, respectively. The enzyme is likely the F_1_F_0_-ATPase based on the usual subunit pattern and the protection against *N,N'*-dicyclohexylcarbodiimide inhibition of ATPase activity by Na^+ ^in a pH-dependent manner. Proteoliposomes reconstituted with the purified enzyme could take up Na^+ ^upon the addition of ATP. The apparent K_*m *_values for this uptake were 3.3 and 0.5 mM for Na^+ ^and ATP, respectively. The mechanism of Na^+ ^transport mediated by Na^+^-stimulated ATPase in *A. halophytica *was revealed. Using acridine orange as a probe, alkalization of the lumen of proteoliposomes reconstituted with Na^+^-stimulated ATPase was observed upon the addition of ATP with Na^+ ^but not with K^+^, Li^+ ^and Ca^2+^. The Na^+^- and ATP-dependent alkalization of the proteoliposome lumen was stimulated by carbonyl cyanide *m *- chlorophenylhydrazone (CCCP) but was inhibited by a permeant anion nitrate. The proteoliposomes showed both ATPase activity and ATP-dependent Na^+ ^uptake activity. The uptake of Na^+ ^was enhanced by CCCP and nitrate. On the other hand, both CCCP and nitrate were shown to dissipate the preformed electric potential generated by Na^+^-stimulated ATPase of the proteoliposomes.

**Conclusion:**

The data demonstrate that Na^+^-stimulated ATPase from *A. halophytica*, a likely member of F-type ATPase, functions as an electrogenic Na^+ ^pump which transports only Na^+ ^upon hydrolysis of ATP. A secondary event, Na^+^- and ATP-dependent H^+ ^efflux from proteoliposomes, is driven by the electric potential generated by Na^+^-stimulated ATPase.

## Background

Most living cells maintain low Na^+ ^ion concentration in the cytoplasm even when the extracellular environment contains a high level of Na^+ ^ions. For cells thriving in a high Na^+ ^ion concentration, there occurs a passive flux of Na^+ ^ions into the cells and this increases the cytoplasmic Na^+ ^concentration [1]. Hypersaline conditions can be deleterious to cells since water is lost to the external medium until osmotic equilibrium is achieved. To adjust the internal osmotic status to enable cells to survive in hypersaline environments, the cells have a mechanism to accumulate compatible low-molecular-weight solutes. The types of these solutes can vary depending on organisms, for example, ectoine in *Chromohalobacter israelensis *(formerly *Bacterium *Ba1) [2], glycine betaine in *Aphanothece halophytica *[3], glucosylglycerol in *Synechocystis *sp. PCC 6803 [4], glycerol in *Saccharomyces cerevisiae *[5], and trehalose in *Desulfovibrio halophilus *[6]. Another mechanism for adaptation to high salinity is extrusion of excessive Na^+ ^ions accumulated in the cytoplasm out of the cells [7]. The well-known mechanism for Na^+ ^extrusion is by Na^+^/H^+ ^antiporters which utilize a H^+ ^gradient generated by H^+^-ATPase catalyzing the movement of Na^+ ^ions across the membrane by exchanging internal Na^+ ^with external H^+ ^[8]. Another mechanism for Na^+ ^transport under high salinity, and at alkaline pH, employs a Na^+^-ATPase or a primary Na^+ ^pump [9]. Na^+^-ATPase plays an important role in the maintenance of the Na^+ ^ion concentration of cells by coupling the hydrolysis of ATP to the translocation of Na^+ ^ions across the cell membrane. Na^+^-ATPase is found in bacteria, fungi, yeast, algae, and halophytic higher plants [10-15]. P-type Na^+^-ATPases were found in *Anabaena *sp. PCC 7120 [16], *Exiguobacterium aurantiacum *[17], *Heterosigma akashiwo *[14] and *Tetraselmis viridis *[18]. V-type Na^+^-ATPases were found in *Caloramator fervidus *[19] and *Enterococcus hirae *[20]. F-type Na^+^-ATPases were found in *Acetobacterium woodii *[21]*, Clostridium paradoxum *[22], *Ilyobacter tartaricus *[23] and *Propionigenium modestum *[24]. However, the information on the ATPase involved in Na^+ ^transport is scarce in cyanobacteria.

*Aphanothece halophytica *is an alkaliphilic halotolerant cyanobacterium that can grow in a wide range of salinity conditions from 0.25-3.0 M NaCl with an alkaline condition as high as pH 11.0 [25]. Intracellular cation levels for *A. halophytica *mainly Na^+ ^and K^+ ^have been reported to be within the range of 260-420 mM depending on the external salinities [26]. Previously we reported the presence of a Na^+^-stimulated ATPase in the plasma membrane of *A. halophytica *[27]. The membrane vesicles could take up Na^+ ^when supplied with ATP. In the halotolerant microalga *Dunaliella maritima*, it was found that Na^+ ^uptake across the plasma membrane by Na^+^-ATPase is electrogenic [28] and the mechanism of Na^+ ^transport by Na^+^-ATPase is suggested to operate as an Na^+ ^uniporter which carries only Na^+ ^ions for transport across the plasma membrane while the transport of H^+^, a counterion, is driven by an electrical membrane potential generated by Na^+^-ATPase [29]. On the contrary, the mechanism of Na^+ ^transport across the plasma membrane of the marine microalga *Tetraselmis viridis *by Na^+^-ATPase is suggested to operate as an Na^+^/H^+ ^exchanger which catalyzes an exchange of Na^+ ^for H^+ ^[30]. In this study, Na^+^-stimulated ATPase from *A. halophytica *was purified and reconstituted into liposomes. The resultant proteoliposomes were used to test whether ATP-dependent Na^+ ^uptake by Na^+^-stimulated ATPase was accompanied with H^+ ^transport. In addition, the nature of coupling between ATP-dependent Na^+ ^transport and H^+ ^flux mediated by Na^+^-stimulated ATPase in *A. halophytica *was investigated.

## Results

### Effect of salinity and pH of growth medium on ATPase activity

**Figure 1:**
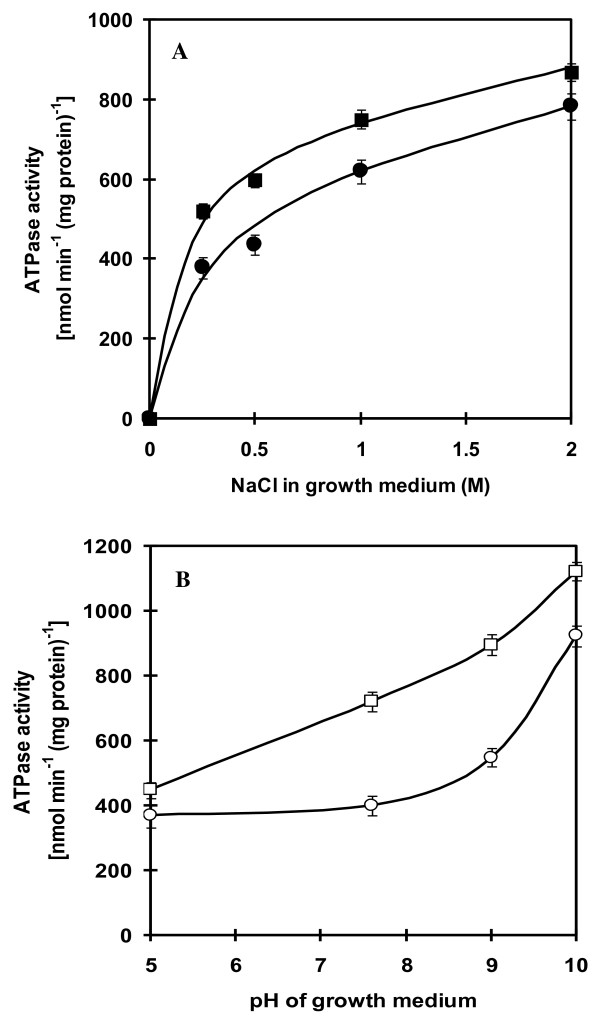
**Effect of NaCl concentration and pH of the growth medium on ATPase activity**. Membrane vesicles were prepared from cells grown at different NaCl concentrations (A) at pH 7.6 (closed circle) or pH 9.0 (closed square), and at different pHs (B) at 0.5 M NaCl (open circle) or 2.0 M NaCl (open square). ATPase activity was assayed in the mixture containing 20 mM Tris-HCl pH 7.6, 5 mM MgCl_2_, 10 mM NaCl and membrane vesicles (30 μg protein). The reaction was started by the addition of 4 mM ATP (Tris salt). All data are the average of three independent experiments with vertical bars representing standard errors.

Since *A. halophytica *is a halotolerant and alkaliphilic cyanobacterium, it is of interest to investigate the changes of ATPase activity in response to changes of NaCl concentration and pH of the growth medium. Increasing NaCl concentration in the growth medium up to 2 M led to a progressive increase of ATPase activity in the membrane vesicles (Figure 1A). Higher ATPase activity was observed in cells grown at high pH than at low pH at all four concentrations of NaCl tested. When the cells were grown at different pH values, ATPase activity in the membrane vesicles was slightly increased upon increasing the pH from 5.0 to 7.6 (Figure 1B). A marked increase of enzyme activity was evident at pH higher than 7.6. Cells grown at 2 M NaCl showed higher enzyme activity than those grown at 0.5 M NaCl at all four pH values tested.

### Purification of ATPase

ATPase is a membrane protein, hence it is necessary to find optimum conditions to solubilize the ATPase from the membranes. Treatment of membrane vesicles with 7 mM sodium cholate for 30 min resulted in the highest yield of ATPase (data not shown).

**Figure 2:**
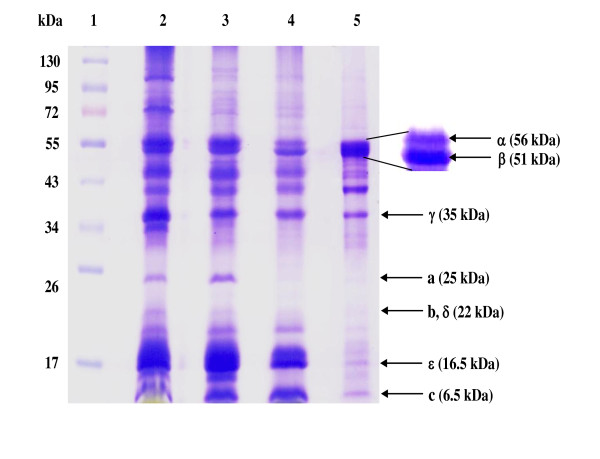
**SDS-PAGE of ATPase from *Aphanothece halophytica***. Samples were resolved on 12% polyacrylamide gels and were stained with Coomassie brilliant blue. Lane 1, PageRuler Prestained Protein Ladder (Fermentas); Lane 2, membrane vesicles; Lane 3, solubilized protein; Lane 4, protein from polyethylene glycol 6000 precipitation; Lane 5, purified ATPase from Superose 6. All F-type ATPase subunits were identified with reference to the previously reported F-type ATPases in *Ilyobacter tartaricus *[22] and *Bacillus *sp. strain TA2.A1 [29]. Samples in all lanes except lane 1 were precipitated with 10% TCA before SDS-PAGE. The contaminating bands were therefore unlikely due to a SDS-stable c ring.

The two-step purification, PEG 6000 precipitation followed by Superose 6 gel filtration, resulted in a 17.5-fold purified enzyme with 6.5% yield (Table 1). It is noteworthy that more than 85% ATPase activity was recovered in the solubilized membrane protein suggesting that nearly all ATPase proteins were solubilized in their native forms. The enzyme was stable up to one month at -20°C. SDS-PAGE analysis (Figure 2) revealed some polypeptide bands which could tentatively be identified as ATPase subunits of an F-type ATPase, namely α (56 kDa), β (52 kDa), γ (35 kDa), a (25 kDa), b (22 kDa), δ (22 kDa), ε (16.5 kDa) and c (6.5 kDa) based on the comparison with the typical mobilities and band pattern of an F-type ATPase subunits from both *Ilyobacter tartaricus *[23] and thermoalkaliphilic *Bacillus *sp. strain TA2.A1 [31]. The subunit c was further characterized by cutting the band on SDS-PAGE (Lane 5) and digesting with trypsin before analysis by liquid chromatography mass spectrometry (LC-MS/MS). The spectra were recorded on an ESI-Q-TOF mass spectrometer (Waters Corporation). The amino acid sequences of the peptides were obtained using Mascot program (http://www.matrixscience.com). One resultant tryptic peptide "ISSGAEGIAR" with the molecular mass of 959.5036, by searching the National Center for Biotechnology Information (NCBI) database, was highly identical to the sequence of F-type ATPase subunit c of *Synechococcus *sp. WH 8102 (UniProtKB Q7U8W9)*, Synechococcus *sp. CC9902 (UniProtKB Q3AZM5), *Synechococcus *sp. PCC 7002 (UniProtKB B1XHZ2) and *Gloeobacter violaceus *(UniProtKB Q7NCR9) with % identity of 90, 90, 80 and 60%, respectively.

**Table 1:** Purification of the ATPase from *Aphanothece halophytica*.

Step	Total protein (mg)	Total activity (unit)^a^	Specific activity (unit mg^-1^)	Purification (fold)	Yield (%)
Membrane vesicles	162.0	4315	26.6	1.0	100.0
Solubilized protein	55.5	3720	67.0	2.5	86.2
PEG 6000 precipitation	4.4	1062	241.4	9.1	24.6
Superose 6	0.6	280	466.7	17.5	6.5

^a ^One unit corresponded to 1 nmol of inorganic phosphate produced per min.

### Catalytic properties of the purified ATPase

**Figure 3:**
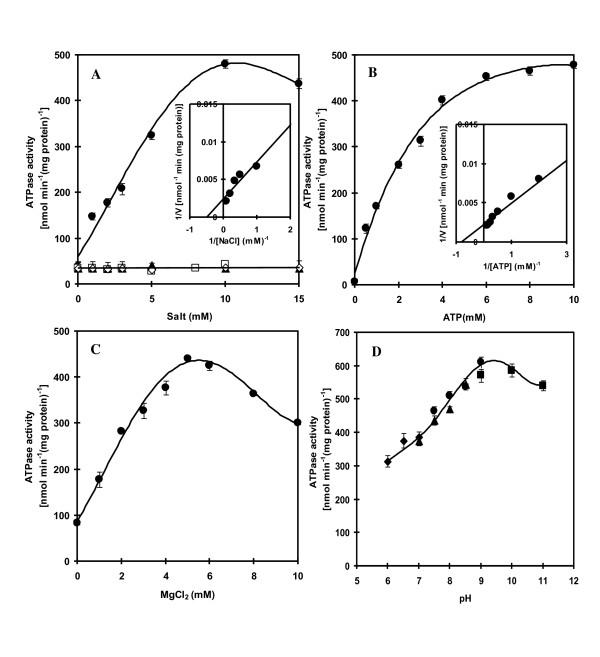
**Dependence of ATPase on cations, ATP, Mg**^**2+ **^**and pH**. In (A), the enzyme activity was measured in the presence of various concentrations of NaCl (closed circle), KCl (open square), LiCl (open rhombus) and CaCl_2 _(closed triangle). Inset shows a double-reciprocal plot of activity versus NaCl concentration. In (B) and (C), the enzyme activity was measured in the presence of various concentrations of ATP and MgCl_2_, respectively. Inset in (B) shows a double-reciprocal plot of activity versus ATP concentration. In (D), the enzyme activity was measured at various pH values using 20 mM Mes-KOH for pH 6.0-7.0 (closed rhombus), 20 mM Hepes-KOH for pH 7.0-8.5 (closed triangle), 20 mM Tris-HCl for pH 7.5-9.0 (closed circle) and 20 mM Glycine-KOH for pH 9.0-11.0 (closed square). All data are the average of three independent experiments with vertical bars representing standard errors.

The activity of ATPase as influenced by various cations was tested and the results are shown in Figure 3A. Increasing Na^+ ^concentration up to 10 mM caused a progressive increase in enzyme activity. K^+^, Li^+^, and Ca^2+ ^had no stimulatory effect on enzyme activity. The apparent *K*_m _value for Na^+ ^as determined from the Lineweaver-Burk plot was 2 mM (Figure 3A, inset). It is noteworthy that only marginal activity was detected in the absence of Na^+ ^accounting for about 10% of the maximum activity. Figure 3B shows that ATPase activity increased as ATP increased and the activity was saturated at about 6 mM ATP. The apparent *K*_m _value for ATP was 1.2 mM (Figure 3B, inset).

**Figure 4:**
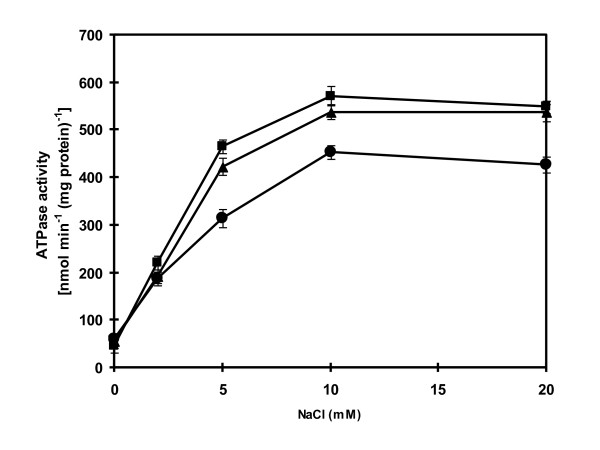
**Effect of increasing concentration of Na**^**+ **^**ions on the ATPase activity**. The ATPase activity was determined in the reaction mixture containing the purified ATPase (30 μg protein), 5 mM MgCl_2 _with either 20 mM Tris-HCl, pH 7.6 (closed circle), 20 mM Tricine-KOH, pH 9.0 (closed square) or 20 mM Glycine-KOH, pH 11.0 (closed triangle) and NaCl concentrations as indicated. The reaction was started by the addition of 4 mM ATP (Tris salt). All data are the average of three independent experiments with vertical bars representing standard errors.

The dependence of ATPase on Mg^2+ ^was also tested. Figure 3C shows that maximal ATPase activity was observed at 5 mM Mg^2+^. The enzyme activity was slightly decreased when the Mg^2+ ^concentration was higher than 5 mM. The pH of the assay medium also influenced the enzyme activity. When the pH was increased from pH 6.0 to pH 9.0, the enzyme activity was increased progressively (Figure 3D). Increasing the pH further to pH 11.0 resulted in a slightly declined enzyme activity. The observation that high activity of ATPase occurred at alkaline pH as high as pH 11.0 prompted us to investigate further the effect of Na^+ ^on the stimulation of ATPase activity at different pH values. Maximal stimulation of ATPase activity occurred at 10 mM NaCl when assayed at pH 7.6, 9.0 and 11.0 (Figure 4). The enzyme was stimulated about 9-fold at pH 7.6 and about 11-fold at both pH 9.0 and 11.0 with 10 mM NaCl.

### Effect of inhibitors on ATPase activity

The protonophore carbonyl cyanide *m*-chlorophenylhydrazone (CCCP), the V-type ATPase inhibitors *N*-ethylmaleimide (NEM), KNO_3 _and KSCN, had no inhibitory effect on ATPase activity (Table 2). Ouabain, an animal Na^+^/K^+^-ATPase inhibitor and orthovanadate, an inhibitor of P-type ATPase, also did not inhibit ATPase activity. Amiloride, a potent inhibitor of many Na^+^-coupled transport systems including Na^+^/H^+ ^antiporters, had no effect on ATPase activity. Sodium azide, an F_1 _inhibitor and *N,N'*-dicyclohexylcarbodiimide (DCCD), an F_0 _inhibitor, inhibited ATPase activity by about 35 and 20%, respectively.

**Table 2:** Effect of inhibitors on ATPase activity of the purified ATPase.

Addition	Residual ATPase activity (%)
None	100
1 mM CCCP	107
0.5 mM NEM	102
50 mM KNO_3_	103
20 mM KSCN	101
1 mM Ouabain	115
0.01 mM Orthovanadate	100
0.2 mM Amiloride	98
5 mM Sodium azide	64
1 mM DCCD	78

ATP hydrolytic activity was assayed in the reaction mixture containing 20 mM Tris-HCl pH 7.6, 5 mM MgCl_2_, 10 mM NaCl and the purified ATPase (30 μg protein). The reaction was started by the addition of 4 mM ATP (Tris salt). Each inhibitor was added to the reaction mixture 10 min before the start of the reaction. One hundred percent activity corresponded to 450 nmol min^-1 ^(mg protein)^-1^.

**Figure 5:**
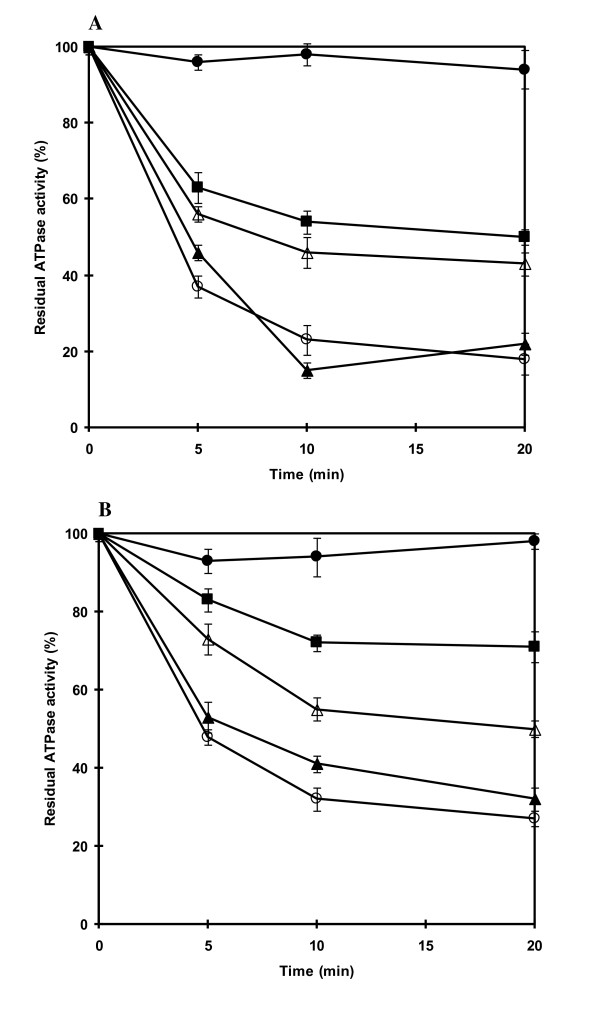
**Protection of ATPase from DCCD inhibition by Na**^**+ **^**at pH 7.6 and pH 9.0**. The purified ATPase was incubated with 200 μM DCCD in either 20 mM Tris-HCl, pH 7.6 (A) or 20 mM Tricine-KOH, pH 9.0 (B). The individual mixtures contained the following additions of NaCl: no NaCl added (open circle), 1 mM NaCl (closed triangle), 10 mM NaCl (open triangle) and 50 mM NaCl (closed square). The residual ATPase activity of samples (30 ug protein) taken at the indicated times was determined in the reaction mixture containing 20 mM Tris-HCl pH 7.6, 5 mM MgCl_2_. The reaction was started by the addition of 4 mM ATP (Tris salt). One hundred percent activity corresponded to 437 nmol min^-1 ^(mg protein)^-1^. Control without DCCD (closed circle). All data are the average of three independent experiments with vertical bars representing standard errors.

The weak inhibition of ATPase activity by DCCD was due to the presence of 10 mM NaCl during the assay of ATPase activity. Indeed, strong inhibition by DCCD in the absence of NaCl occurred after 20 min yielding 80 and 70% inhibition at pH 7.6 and pH 9.0, respectively (Figures 5A, B). The presence of 1 mM NaCl during incubation with DCCD had no (at pH 7.6) or little (at pH 9.0) protection against DCCD inhibition. However, increasing the concentration of NaCl led to the reduction of the inhibition by DCCD on ATPase activity for both conditions at pH 7.6 and 9.0. Stronger protection was observed at pH 9.0 than at pH 7.6. Treatment with DCCD for 20 min in the presence of 50 mM NaCl resulted in approximately 30 and 50% inhibition at pH 9.0 and 7.6, respectively. The presence of 20 mM LiCl or KCl during incubation with DCCD afforded no protection against DCCD inhibition (data not shown).

### Catalytic properties of proteoliposomes

The purified Na^+^-ATPase from *A. halophytica *reconstituted into proteoliposomes by a freeze-thaw/dilution method was characterized. The tests for the effects of cations, ATP, Mg^2+^, pH and inhibitors on ATPase activity of reconstituted proteoliposomes yielded similar results to those of the purified enzyme (data not shown).

### Transport of Na^+ ^into proteoliposomes

**Figure 6:**
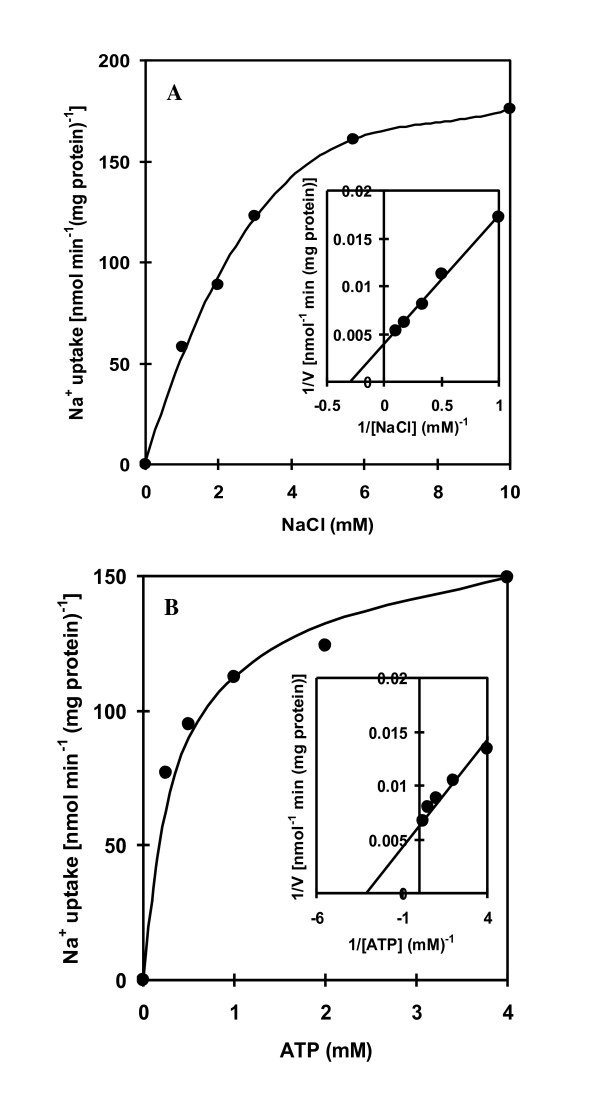
**Dependence of Na**^**+ **^**uptake on Na**^**+ **^**and ATP**. Na^+ ^uptake into reconstituted proteoliposomes was measured in 20 mM Tris-HCl pH 7.6 containing 5 mM MgCl_2 _and at various concentrations of ^22^NaCl **(**80 μCi mmol^-1^) (A) and ATP (B). The uptake reaction was started by the addition of 4 mM ATP (Tris salt). Insets in (A) and (B) show double-reciprocal plots of Na^+ ^uptake versus NaCl and ATP concentration, respectively.

The transport of Na^+ ^into proteoliposomes reconstituted with ATPase increased with increasing concentration of NaCl with an apparent saturation at about 10 mM NaCl (Figure 6A) and the apparent *K*_m _value for Na^+ ^of 3.3 mM (Figure 6A, inset). Moreover, Na^+ ^accumulated in the proteoliposomes after the addition of ATP while no significant uptake of Na^+ ^was detected in the absence of ATP (Figure 6B). An increase in ATP concentration resulted in an increase of Na^+ ^transport reaching saturation at about 4 mM ATP. The apparent *K*_m _value for ATP was estimated to be 0.5 mM (Figure 6B, inset).

**Figure 7:**
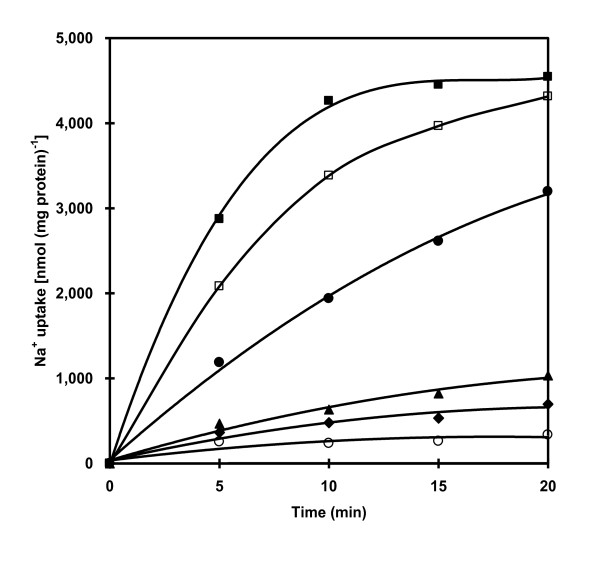
**Time course of Na**^**+ **^**uptake into reconstituted proteoliposomes**. Na^+ ^uptake into proteoliposomes was measured in 20 mM Tris-HCl pH 7.6 containing 5 mM MgCl_2 _and 5.7 mM ^22^NaCl **(**80 μCi mmol^-1^) at times indicated in the presence (closed circle) and absence (open circle) of 4 mM ATP and in the presence of 4 mM ATP with 0.05 mM CCCP (closed square), 100 mM KNO_3 _(open square), 0.1 mM monensin (closed rhombus), 0.01 mM gramicidin D (closed triangle). Effectors were added at 10 min before starting the reaction with ATP.

The time course of Na^+ ^uptake into proteoliposomes reconstituted with purified ATPase is shown in Figure 7. In the absence of ATP, Na^+ ^uptake was very low but upon the addition of ATP the uptake was significantly accelerated. The ATP-dependent Na^+ ^uptake was strongly inhibited by gramicidin D and monensin while a protonophore CCCP and the permeant anion nitrate stimulated the uptake of Na^+^.

### Detection of H^+ ^efflux from proteoliposomes

**Figure 8:**
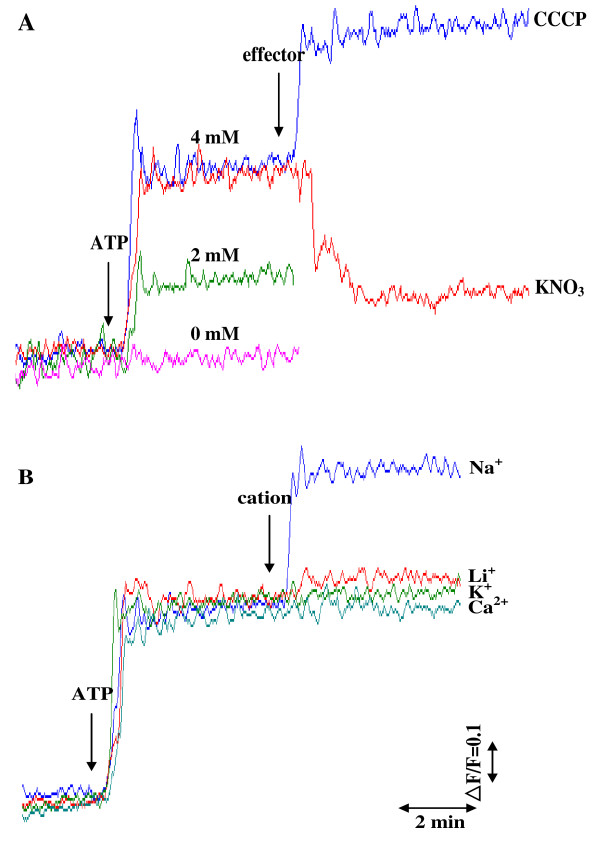
**Changes in **Δ**pH of proteoliposome lumen detected with the **Δ**pH probe acridine orange**. (A) Effect of ATP concentration and effectors (CCCP and permeant anion NO^-^_3_) on alkalization of proteoliposome lumen. 100 mM NaCl was initially present in the reaction mixture containing 0.5 M sorbitol, 10 mM Hepes-Tris buffer pH 7.6, 1 μM acridine orange and proteoliposomes (25 μg protein). After 15 min incubation of proteoliposomes in the mixture, the reaction was run by the addition of ATP at various concentrations and effectors (CCCP at 12 μM or KNO_3 _at 100 mM) as shown by the arrows. **(B) **Effect of cations on alkalization of proteoliposome lumen. NaCl was initially present in the reaction mixture at the concentration of 8 mM. The reaction was run by the addition of ATP at 4 mM and cations (chloride salts) at 100 mM as shown by the arrows.

In order to investigate whether Na^+ ^uptake into the proteoliposomes is accompanied with H^+ ^efflux from the proteoliposomes, alkalization of the proteoliposome lumen was detected using the ΔpH probe acridine orange with a p*K*_*a *_of 10.45. Addition of ATP to the reaction medium containing NaCl at 100 mM initiated H^+ ^efflux from the proteoliposome lumen to the outer medium (Figure 8A). This H^+ ^efflux was ATP concentration-dependent. The protonophore CCCP induced ATP-dependent lumen alkalization while the permeant anion nitrate suppressed ATP-dependent lumen alkalization. When Na^+ ^in the reaction medium was replaced with K^+^, Li^+ ^and Ca^2+^, the ATP-dependent alkalization of the proteoliposome lumen was not observed (Figure 8B).

### Detection of membrane potential

**Figure 9:**
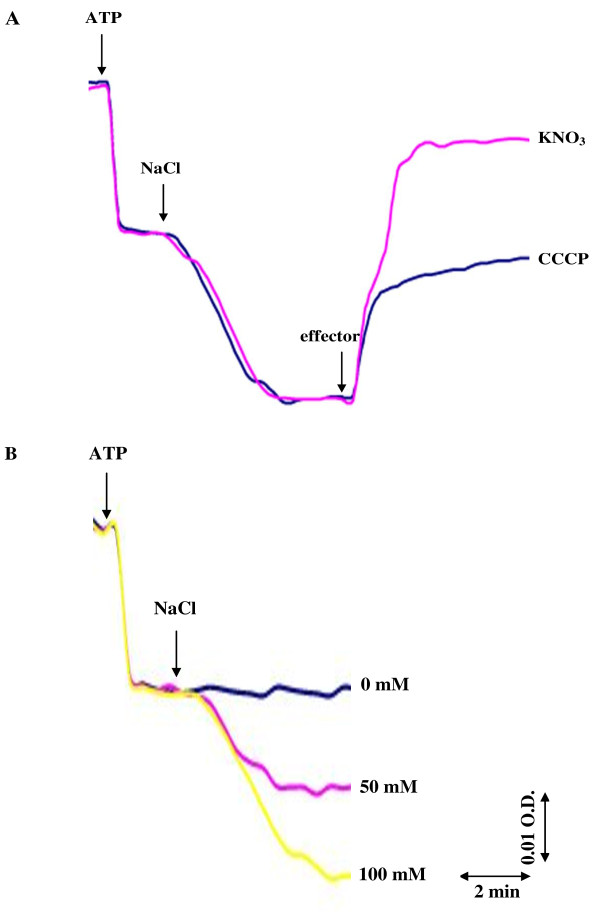
**Changes in membrane potential across proteoliposome detected with voltage-sensitive probe oxonol VI**. (A) Effect of CCCP and the permeant anion NO^-^_3 _on the membrane potential across the proteoliposome built up in the presence of ATP and Na^+^. 8 mM NaCl was initially present in the reaction mixture containing 0.4 M sucrose, 20 mM Hepes-Tris buffer pH 7.6, 1 mM MgSO_4_, 3 μM oxonol VI and proteoliposomes (25 μg protein). The reaction was initiated by adding ATP at 4 mM, NaCl at 100 mM, CCCP at 12 μM or KNO_3 _at 100 mM as shown by the arrows. (B) Effect of NaCl concentration on the membrane potential across the proteoliposome. NaCl was initially present in the reaction mixture at the concentration of 8 mM. The reaction was initiated by adding ATP at 4 mM and NaCl at various concentrations as shown by the arrows.

To investigate the nature of coupling of Na^+ ^and H^+ ^fluxes upon the addition of ATP to proteoliposomes reconstituted with the purified ATPase, the role of membrane potential in the ATP-dependent H^+ ^translocation was studied with a voltage-sensitive probe oxonol VI. Figure 9A shows the generation of membrane potential (positive inside the proteoliposome lumen) upon the addition of ATP to the reaction medium containing 8 mM NaCl. The generation of membrane potential was further enhanced when 100 mM NaCl was added. These results indicated that the membrane potential was built up by the operation of a Na^+^-stimulated ATPase. On the contrary, both the protonophore CCCP and the permeant anion nitrate collapsed the membrane potential built up by Na^+^-stimulated ATPase. Figure 9B shows that the increase in membrane potential generation was Na^+ ^concentration-dependent.

## Discussion

There have been studies of ATPases associated with various cations such as Ca^2+^, Cu^2+^, K^+ ^and Mg^2+ ^in a number of cyanobacteria [32-35]. However, ATPase involved in Na^+ ^transport has not been directly demonstrated experimentally in cyanobacteria. In this study, we focused on the involvement of ATPase in Na^+ ^homeostasis in the alkaliphilic halotolerant cyanobacterium *A. halophytica*. High ATPase activity detected in membrane vesicles of cells grown under high salinity and high pH conditions (Figure 1A, B) suggested that ATPase from *A. halophytica *plays a role in the response of cells against an increase in salinity and pH. Similar observations were found in *Streptococcus faecalis*, *Tetraselmis viridis*, and *Heterosigma akashiwo *[36,37]. Furthermore, we also found that *A. halophytica *requires Na^+ ^for its growth. The presence of gramicidin D, an ionophore that dissipates Na^+^-gradients, in the growth medium resulted in the cessation of cell growth (data not shown). These results are in agreement with those previously reported in a facultative anaerobic alkaliphile M-12 (*Amphibacillus *sp.) which utilized sodium motive force (ΔμNa^+^) generated by a sodium pump for active transport of solutes while other alkaliphiles produced ΔμNa^+ ^by a Na^+^/H^+ ^antiporter which is a secondary transport system for Na^+ ^[15,38,39].

To investigate Na^+ ^transport in relation to ATPase activity in *A. halophytica*, the ATPase was purified and was tentatively classified as an F-type ATPase. This is based on the results that its activity was inhibited by azide and DCCD, which are inhibitors of F_1_F_0_-ATPase but not by orthovanadate and nitrate which are inhibitors of P-type and V-type ATPases, respectively (Table 2). DCCD is believed to inhibit F-type ATPase due to the binding of DCCD to a highly conserved carboxyl residue in the c subunit of the F_0 _portion [40]. The band pattern of the purified enzyme analyzed by SDS-PAGE as shown in Figure 2 was comparable to the typical mobilities and band pattern of F-type ATPase subunits from both *Ilyobacter tartaricus *[23] and a thermoalkaliphilic *Bacillus *sp. strain TA2.A1 [31]. The polypeptide bands could tentatively be identified as ATPase subunits of an F-type ATPase, namely: α (56 kDa), β (52 kDa), γ (35 kDa), a (25 kDa), b (22 kDa), δ (22 kDa), ε (16.5 kDa) and c (6.5 kDa). Moreover, the band tentatively identified as F-type ATPase subunit c of *A. halophytica *was confirmed by LC-MS/MS analysis. One of the obtained sequences after trypsin digestion namely "ISSGAEGIAR" was found to be highly identical to the partial sequence of F-type ATPase subunit c of at least four strains of cyanobacteria, namely *Synechococcus *sp. WH 8102*, Synechococcus *sp. CC9902, *Synechococcus *sp. PCC 7002 and *Gloeobacter violaceus. *Overall, the results from the inhibitor effects, and the typical subunits band pattern as well as the protection by Na^+ ^against DCCD inhibition of ATPase activity (Figure 5), suggest that the ATPase from *A. halophytica *is likely a member of the F-type ATPases. Immunological studies as well as the analysis of the N-termini of different subunits can help establish the actual class of ATPase from *A. halophytica. *Further characterization of the Na^+^-stimulated ATPase in *A. halophytica *by genetic manipulations is now under way.

The purified ATPase from *A. halophytica *was reconstituted into liposomes to investigate the possible role of Na^+^-stimulated ATPase on the transport of Na^+^. The reconstituted ATPase showed catalytic properties, namely *K*_m_'s of Na^+ ^and ATP identical to those of the purified ATPase. Moreover, an increase in the concentration of NaCl and ATP led to an increase in Na^+ ^uptake by the proteoliposomes (Figure 6A, B). These results suggest the presence of an ATP-dependent Na^+ ^pump in the proteoliposomes. The substrate ATP was hydrolyzed by ATPase to provide a driving force for Na^+ ^uptake. A previous report demonstrated the function of Na^+^-ATPase as a Na^+ ^pump in the plasma membrane of the marine alga *Heterosigma akashiwo *based on the similarity of the kinetic properties of Na^+ ^transport and Na^+^-ATPase activity [14]. In *A. halophytica*, the apparent *K*_m _value of Na^+ ^transport for Na^+ ^and ATP were 3.3 and 0.5 mM, respectively. These values are close to the apparent *K*_m _values of Na^+^-stimulated ATPase activity for Na^+ ^(2 mM) and ATP (1.2 mM). This indicates that the purified ATPase from *A. halophytica *is indeed a Na^+ ^pump. The uptake of Na^+ ^into proteoliposomes was abolished by gramicidin D and monensin whereas a protonophore CCCP and the permeant anion nitrate had a stimulatory effect on Na^+ ^uptake (Figure 7). These results suggest the operation of electrogenic Na^+ ^transport by Na^+^-stimulated ATPase in *A. halophytica*. Moreover, ATP-dependent Na^+ ^uptake by proteoliposomes was a primary and not a secondary event, i.e. the transport was not catalyzed by a Na^+^/H^+ ^antiporter or other secondary events driven by proton potential.

As observed in Figure 8A, Na^+ ^uptake by ATPase is associated with H^+ ^efflux in *A. halophytica*. Previously, the marine alga *Tetraselmis viridis *was shown to contain Na^+^-ATPase capable of translocating Na^+ ^into plasma membrane vesicles which was accompanied by H^+ ^efflux with the consequence of the alkalization of the vesicle lumen [41]. In this study, we attempted to elucidate the mechanism of Na^+^- and H^+^-transport by Na^+^-stimulated ATPase reconstituted into liposomes. Two possible mechanisms of Na^+^-transport can be hypothesized [30]. Mechanism 1 proposes that the Na^+^-stimulated ATPase operates as a uniporter catalyzing only Na^+ ^transport across proteoliposomes, subsequently, H^+ ^transport is driven by the membrane potential generated by Na^+^-stimulated ATPase. Mechanism 2 proposes that the Na^+^-stimulated ATPase operates as an antiporter catalyzing an exchange of Na^+ ^for H^+^.

To distinguish between these two mechanisms, we studied the role of membrane potential in ATP-dependent H^+ ^translocation. The dissipation of membrane potential would result in complete suppression of H^+ ^efflux, if the membrane potential across the proteoliposome generated by Na^+^-stimulated ATPase were the only driving force for H^+ ^extrusion. On the other hand, if H^+ ^efflux occurred directly via the Na^+^- stimulated ATPase as an antiporter by exchanging Na^+ ^for H^+^, the dissipation of the membrane potential would not suppress H^+ ^efflux [41]. A permeant anion, nitrate, that generally penetrates easily across biological membranes down its electrochemical gradient is often used as a charge-compensating anion to abolish membrane potential (positive inside) across proteoliposomes during the measurements of ATP-driven H^+ ^translocation. If nitrate inhibits the ATP-dependent alkalization of the proteoliposome lumen and promotes both dissipation of membrane potential and ATP-dependent Na^+ ^uptake, it can be concluded that ATP- and Na^+^-dependent H^+ ^efflux from proteoliposomes is driven by the membrane potential generated by Na^+^-ATPase. Hence, Na^+^-ATPase operates as a uniporter carrying only Na^+^, and H^+ ^is not involved in the catalytic cycle of this enzyme. On the contrary, if nitrate increases both ATP-dependent alkalization of the proteoliposome lumen and Na^+ ^uptake while membrane potential is dissipated, it can be concluded that Na^+^-stimulated ATPase operates as an antiporter catalyzing Na^+^/H^+ ^exchange.

Our results are consistent with mechanism 1 by which the purified Na^+^-stimulated ATPase operates as a uniporter. The ATP-dependent Na^+ ^uptake in proteoliposomes was accelerated in the presence of CCCP and nitrate (Figure 7) and both agents dissipated membrane potential generated by Na^+^-stimulated ATPase (Figure 9A). CCCP stimulated ATP-dependent alkalization of proteoliposomes while nitrate inhibited this alkalization (Figure 8A). Only Na^+ ^but not K^+^, Li^+ ^and Ca^2+ ^promoted H^+ ^efflux (Figure 8B). The results indicate that H^+ ^efflux from proteoliposomes is driven by the membrane potential generated by Na^+^-stimulated ATPase and is specific for Na^+^. Moreover, this dependence of lumen alkalization on Na^+ ^coincided with the requirement of Na^+ ^but not K^+^, Li^+ ^and Ca^2+ ^on ATPase activity (Figure 3A). Therefore, we can conclude that 1) Na^+^- stimulated ATPase from *A. halophytica *operates as a uniporter which takes up only Na^+ ^while H^+ ^is counterion and 2) ATP-dependent lumen alkalization occurred as a result of the operation of the Na^+^-stimulated ATPase. H^+ ^efflux does not occur directly via Na^+^-stimulated ATPase but is driven by the membrane potential generated during Na^+^-stimulated ATPase operation. The operation of Na^+^-stimulated ATPase as a Na^+ ^uniporter has been previously reported in the halotolerant microalga *Dunaliella maritima *[28] while Na^+^-translocating ATPase in the marine microalga *Tetraselmis viridis *operates as a Na^+^/H^+ ^exchanger [30].

Despite the fact that several potential Na^+^-ATPases have been implicated in cyanobacteria, the existence of a Na^+^-ATPase responsible for Na^+ ^movement has not been experimentally demonstrated. Recent studies in the genome of *Synechocystis *sp. PCC 6803 showed that the disruption of a gene cluster encoding a putative Na^+^-ATPase subunit led to high NaCl sensitivity of the mutant suggesting the role of Na^+^-ATPase in salt resistance [42]. Brown *et al*. [43] reported that NaCl could stimulate light-supported generation of membrane potential in the marine cyanobacterium *Oscillatoria brevis*. They further suggested that *O. brevis *might possess a light-dependent primary Na^+ ^pump in the cytoplasmic membrane but it is not clear what kind of a primary Na^+ ^pump operates in the cytoplasmic membrane of *O. brevis*. *Synechococcus *R-2 PCC 7942 was postulated to have a primary Na^+ ^pump extruding Na^+ ^in the light and dark utilizing Na^+ ^motive force [1]. Our study in *A. halophytica *is the first report that experimentally demonstrates the involvement of Na^+^-stimulated ATPase in Na^+ ^transport in cyanobacteria using a purified protein incorporated into liposomes.

## Conclusion

The purified Na^+^-stimulated ATPase from *A. halophytica *is likely a member of the F_1_F_0 _ATPase family. The uptake of Na^+ ^into proteoliposomes is mediated by this enzyme upon ATP hydrolysis. The transport of Na^+ ^is electrogenic and operates via a uniport mechanism with H^+ ^countertransport as a secondary event energized by the membrane potential generated by the operation of Na^+^-stimulated ATPase.

## Methods

### Growth of organism

*Aphanothece halophytica *cells were grown photoautotrophically in BG11 medium supplemented with 18 mM NaNO_3 _and Turk Island salt solution as described previously [44]. Cells were grown in a 2-L Erlenmeyer flask containing 1 L of medium at 30°C under continuous illumination by cool white fluorescence tubes of 25 μmol photon m^-2 ^s^-1^. The aeration of the culture was provided in the form of air bubbles by an air pump.

### Preparation of membrane vesicles and ATPase purification

Cells at exponential growth phase were harvested and washed with 20 mM Tris-HCl pH 7.6 containing 1.0 M sucrose. The collected cells were resuspended in extraction buffer (20 mM Tris-HCl pH 7.6, 1 mM DTT, 4 mM benzamidine and 5 mM MgCl_2_) with ratio (g ml^-1^) of 1.0-2.0. After homogenization, the cells were disrupted by two passages through a French pressure cell at 400 kPa. Unbroken cells and large debris were removed by centrifugation at 9,800 *g *for 15 min. The membrane vesicles were sedimented by centrifugation at 100,000 *g *for 30 min and resuspended in 50 mM Hepes-KOH pH 7.0 to a final protein concentration of 1 mg ml^-1^. The ATPase was solubilized by incubation of the membrane vesicles with sodium cholate at a final concentration of 7 mM. After 30 min with occasional mixing at 0°C, the solubilized membrane proteins were isolated by centrifugation at 100,000 *g *for 1 h. The solubilized ATPase was supplied with MgCl_2 _to a final concentration of 50 mM. Contaminating proteins were precipitated with PEG 6000 (2% w/v). The precipitate was removed by centrifugation at 40,000 *g *for 15 min. PEG 6000 (7% w/v) was added to the supernatant to precipitate the ATPase. The pellet was collected by centrifugation at 40,000 *g *for 15 min and dissolved in 1 ml of 50 mM Tris-HCl pH 7.6 containing 1 mM DTT and 0.1 mM benzamidine. Insoluble material was removed by centrifugation at 3,800 *g *for 10 min and the supernatant containing ATPase was subject to gel chromatography with a superose 6 HR10/30 column pre-equilibrated with 50 mM Tris-HCl pH 7.6, 150 mM KCl, 5 mM MgCl_2 _and 0.4 mM sodium cholate. The active ATPase fractions were pooled, dialyzed, concentrated and kept at -20°C.

### Determination of ATPase activity

The ATPase activity was assayed by measuring the release of inorganic phosphate resulting from the hydrolysis of ATP [45]. The reaction mixture (1 ml) contained 20 mM Tris-HCl pH 7.6, 5 mM MgCl_2_, 10 mM NaCl, and ATPase (30 μg protein). The reaction was started by the addition of 4 mM ATP (Tris salt).

### Preparation of reconstituted proteoliposomes

Reconstituted proteoliposomes were prepared as described by Neumann *et al*. [23] with slight modification. A suspension of 60 mg phosphatidylcholine (Sigma; Type II S) in 1.9 ml of 50 mM Tris-HCl pH 7.6, 1 mM MgCl_2_, 1 mM DTT and 5 mM sodium cholate was sonicated until the suspension was clear. The detergent was used as a disaggregating agent to aid membrane protein reconstitution. Purified ATPase (0.1 ml; 0.3 mg protein) was added to the suspension and the mixture was incubated at 25°C for 10 min with occasional shaking, then frozen in liquid nitrogen and thawed at 0°C. The proteoliposomes were sonicated twice for 5 s each and diluted 200-fold with 50 mM Tris-HCl pH 7.6. The proteoliposomes were collected by centrifugation at 100,000 *g *for 60 min and resuspended in 0.3 ml of 5 mM Tris-HCl pH 7.6 containing 1 mM MgCl_2_.

### Determination of Na^+ ^uptake by proteoliposomes

The proteoliposomes (30 μg protein) were suspended in 60 μl of 20 mM Tris-HCl pH 7.6 containing 5 mM MgCl_2 _and 5.7 mM ^22^NaCl (80 μCi mmol^-1^). The uptake reaction was started by the addition of 4 mM ATP (Tris salt). After equilibration for 30 min, 50 μl of the reaction mixture was filtered through a 0.2 μm cellulose acetate membrane. The membrane filter was washed once with 1 ml of 20 mM Tris-HCl pH 7.6 before measuring the radioactivity with a liquid scintillation counter. Ionophores and inhibitors were added 10 min before starting the reaction with ATP.

### Detection of H^+ ^efflux from proteoliposomes

H^+ ^efflux from proteoliposomes was determined as ATP-dependent alkalization of the proteoliposome lumen. The assay was performed at room temperature by monitoring the changes in fluorescence intensity of the ΔpH probe acridine orange with a fluorescence spectrophotometer set at 493 nm (excitation) and 525 nm (emission) [46,47]. The assay was performed in 1 ml of a reaction mixture containing 0.5 M sorbitol, 10 mM Hepes-Tris buffer pH 7.6, 1 μM acridine orange, 100 mM NaCl, and proteoliposomes (25 μg protein). After 15 min incubation of proteoliposomes in the medium, the reaction was initiated by the addition of ATP (Tris salt) at 4 mM.

### Detection of membrane potential

ATP-dependent formation of the membrane potential across proteoliposomes was detected by monitoring the differential absorbance changes (621/582 nm) of the membrane potential probe oxonol VI using a dual wavelength spectrophotometer as described by Popova *et al*. [28] with slight modification. The reaction mixture (1 ml) contained 0.4 M sucrose, 20 mM Hepes-Tris buffer pH 7.6, 1 mM MgSO_4_, 3 μM oxonol VI and proteoliposomes (25 μg protein). The reaction mixture was incubated for 15 min at room temperature. The generation of membrane potential was initiated by supplementing the reaction mixture with ATP (Tris salt) and NaCl at final concentrations of 4 and 100 mM, respectively.

### Determination of protein

Protein contents were determined by the protein-dye binding method of Bradford [48] using bovine serum albumin as standard.

## List of abbreviations

ATPase: adenosine 5'-triphosphatase; CCCP: carbonyl cyanide *m*-chlorophenylhydrazone; DCCD: *N,N'*-dicyclohexylcarbodiimide; DTT: dithiothreitol; NEM: *N*-ethylmaleimide; PEG 6000: polyethylene glycol 6000; ΔpH: trans membrane pH gradient.

## Authors' contributions

AI conceived the project, designed the experiments, provided advice, and wrote the manuscript. KS designed and performed the experiments, prepared figures and tables, and partially wrote the manuscript. Both authors read and approved the final manuscript.
